# Morning Glory Anomaly With Serous Macular Detachment

**DOI:** 10.7759/cureus.57817

**Published:** 2024-04-08

**Authors:** Kalpita B Goli, Renu Magdum, Shreya Gandhi, Jhimli Ta

**Affiliations:** 1 Ophthalmology, Dr. D. Y. Patil Medical College, Hospital, and Research Centre, Pune, IND

**Keywords:** optic disc coloboma, subretinal fluid, acetazolamide, serous macular detachment, morning glory syndrome

## Abstract

The morning glory (MG) disc anomaly is a congenital excavation of the posterior globe involving the optic disc, with a distinct appearance reminiscent of the MG flower. Various intracranial and ocular associations with MG have been documented. Conditions such as trans-sphenoidal encephalocele and hypoplasia of the intracranial vasculature have been observed in association with this anomaly. In this report, we present a case of MG optic disc anomaly accompanied by serous macular detachment.

## Introduction

The morning glory (MG) disc anomaly is a congenital excavation of the posterior globe involving the optic disc, with a distinct appearance reminiscent of the MG flower. Various intracranial and ocular associations with MG have been documented. Conditions such as trans-sphenoidal encephalocele and hypoplasia of the intracranial vasculature have been observed in association with this anomaly. In this report, we present a case of MG optic disc anomaly accompanied by serous macular detachment.

## Case presentation

**Figure 1:**
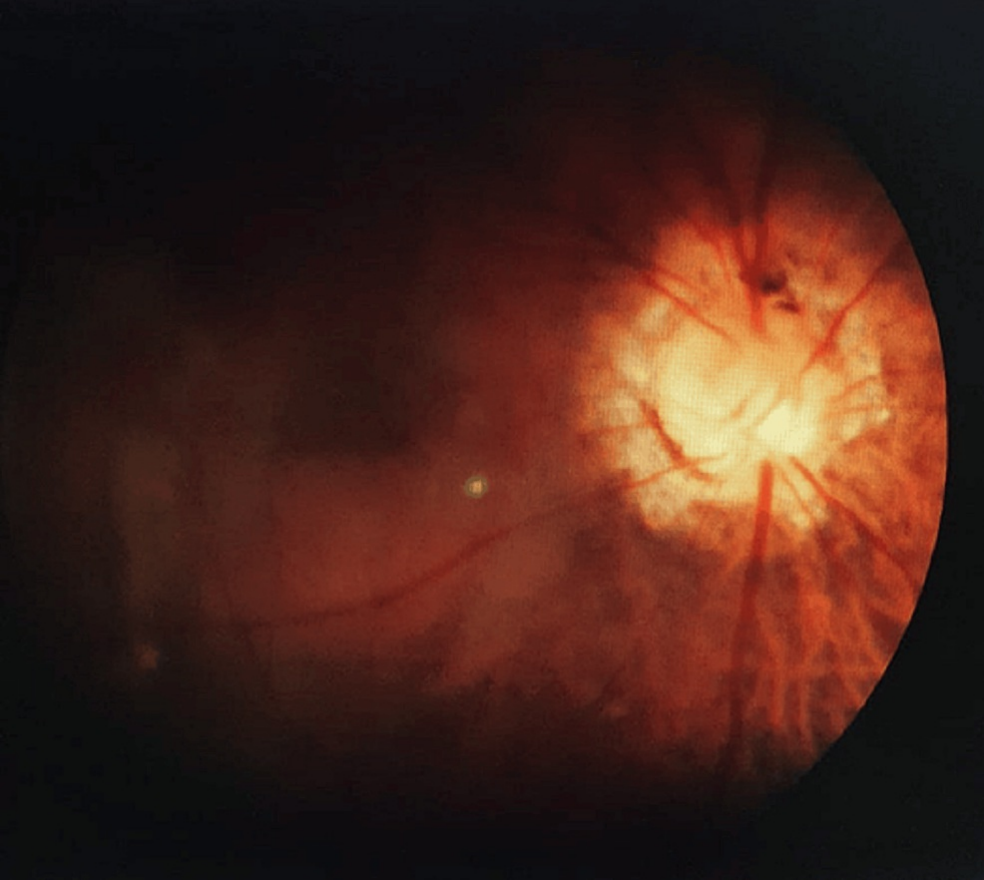
Fundus image (right eye) showing the classical morning glory appearance of the optic disc

**Figure 2:**
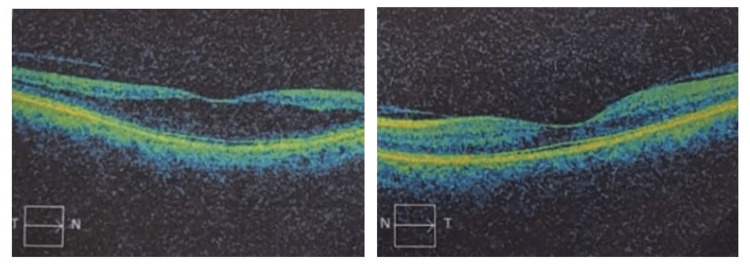
SD-OCT image: the right eye shows serous macular detachment with increased foveal thickness with the left eye being normal SD-OCT: spectral-domain optical coherence tomography

**Figure 3:**
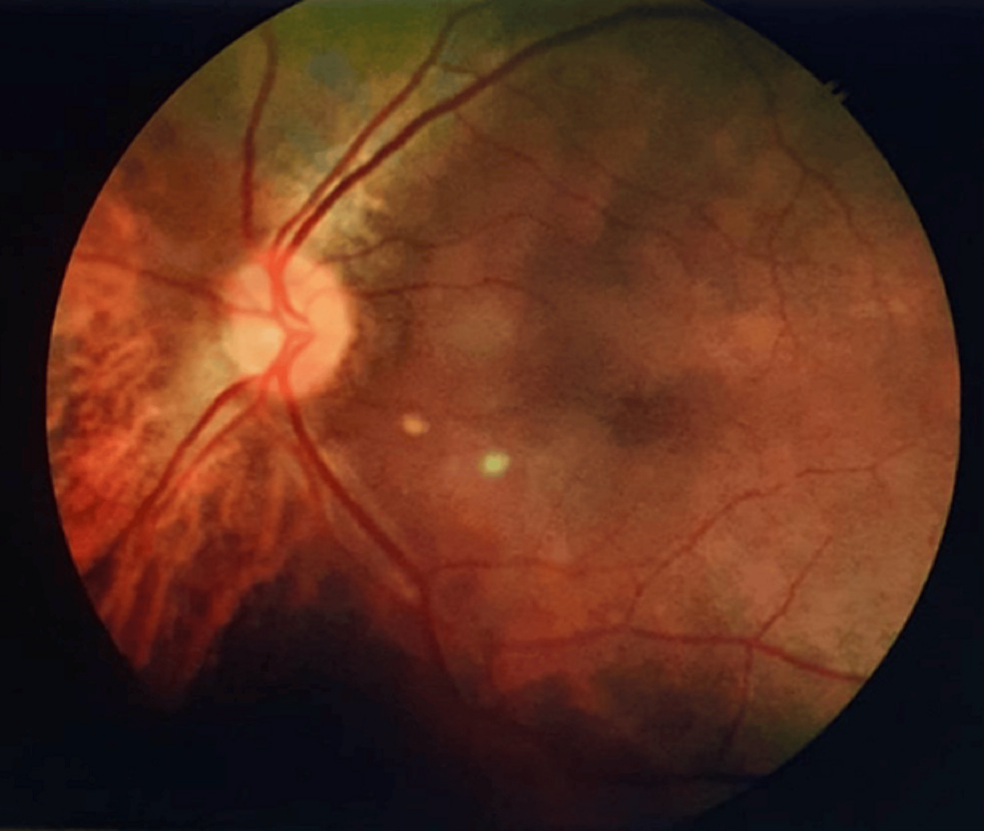
Fundus image (left eye) with normal optic disc

A 55-year-old woman visited the ophthalmology outpatient department (OPD), reporting reduced vision in her right eye (RE). She had experienced poor vision in that eye since birth but noted a further decrease in the past few months. Upon examination, her vision in the RE was recorded as 6/60 on the Snellen chart and did not improve with lenses. The slit-lamp examination revealed a senile cataract. Fundus examination of the RE exhibited a typical MG disc with some macular elevation (Figure 1). Spectral-domain optical coherence tomography (SD-OCT) confirmed the presence of serous macular detachment with a central foveal thickness of 473 microns (Figure 2). The left eye appeared normal (Figure 3), except for an early cataract, with a vision of 6/12 on the Snellen chart.

The patient was started on oral acetazolamide, 250 mg twice a day, for one month. Subsequently, her visual acuity improved to 6/24. A repeat SD-OCT showed a normalized central foveal thickness of 270 microns.

## Discussion

The MG disc anomaly is recognized as distinct from optic disc colobomas, marked by its sporadic occurrence, absence of association with iris or retinal colobomas, and systemic associations. This unilateral condition often leads to poor vision in one eye from birth and is found to be twice as common in females [1].

Various associations with MG syndrome have been reported, including moyamoya syndrome [2]. Other documented associations include persistent hyaloid tissue, lid hemangiomas, retinal gliosis, retinoschisis, peripapillary contractile staphyloma, optic disc pit, lens coloboma, glaucoma, and Duane's retraction syndrome [3,4].

Retinal detachment (RD) is a potential complication in up to one-third of patients with MG syndrome, and spontaneous resolution of these detachments has been observed [5]. The subretinal fluid may originate from either the cerebrospinal fluid (CSF) or the vitreous cavity, with tractional forces also being implicated. These detachments tend to recur but can also resolve spontaneously [3].

In a retrospective case review series conducted at the Department of Ophthalmology, Columbia University Medical Center, two eyes with MG anomaly were evaluated between 2006 and 2011 [3]. The first case involved a 44-year-old woman who presented with MG optic discs in both eyes. Over a follow-up period of seven years, she experienced intermittent episodes of blurred vision in both eyes. These episodes were diagnosed as retinoschisis and serous RDs, which spontaneously improved over variable periods. In each eye, there appeared to be an oval retinal break within the thin, detached retina, which was pulled into the excavation of the MG optic disc.

The second case involved a 49-year-old woman with a RE-MG anomaly who developed an RD. The patient was treated with pneumatic retinopexy, followed by laser treatment and vitrectomy. Acetazolamide is known to increase the pump activity of the retinal pigment epithelium (RPE). Such cases are challenging to manage surgically. Its use has been reported in Sturge-Weber syndrome [6].

Acetazolamide enhances choroidal blood flow and improves the pumping function of the RPE, which aids in draining the subretinal fluid into the choroid. Thus, in eyes with RD, the RPE dehydrates the subretinal space, while acetazolamide significantly enhances the absorption of subretinal fluid [7,8]. Gonzalez studied the effectiveness of oral acetazolamide at a dosage of 0.375 g daily in three divided doses in 38 patients [9]. He observed a reduction in metamorphopsia in all cases, a stabilization or even improvement of visual acuity, and a resorption of serous RD, as confirmed by the decreased pooling of fluorescein in angiographic examinations.

In our case, we administered 250 mg of oral acetazolamide twice daily for a month. This treatment improved visual acuity and significantly reduced central foveal thickness, demonstrating that even a lower dose of acetazolamide can be effective.

## Conclusions

MG syndrome, a rare condition, may have vision-threatening complications like serous RD which may be amenable to be managed medically. Management often involves monitoring for associated conditions like RD and amblyopia, and treatment may include surgical intervention in some cases. Early detection and intervention are crucial for optimizing visual outcomes.
